# Caught in vicious circles: a perspective on dynamic feed-forward loops driving oxidative stress in schizophrenia; Response to “Adaptive changes to oxidative stress in schizophrenia by Lena Palaniyappan”

**DOI:** 10.1038/s41380-022-01553-3

**Published:** 2022-04-28

**Authors:** Michel Cuenod, Pascal Steullet, Jan-Harry Cabungcal, Daniella Dwir, Ines Khadimallah, Paul Klauser, Philippe Conus, Kim Q. Do

**Affiliations:** 1 Center for Psychiatric Neuroscience, Department of Psychiatry, Lausanne University Hospital (CHUV), Prilly, Lausanne, Switzerland; 2 Service of Child and Adolescent Psychiatry, Department of Psychiatry, Lausanne University Hospital, Prilly, Lausanne, Switzerland; 3 Service of General Psychiatry, Department of Psychiatry, Lausanne University Hospital, Prilly, Lausanne, Switzerland

**Keywords:** Schizophrenia, Neuroscience

## To the Editor:

We thank Dr. Lena Palaniyappan for his thoughtful comment [1] to our recent review [2] and for giving us this opportunity to clarify some points in order to avoid misunderstandings.

The proposal that major pathological factors in psychosis (mitochondria, inflammation, Glu/NMDAR,) form a circularly self-entertaining and generalizing mechanism with the redox system is based primarily on observations made experimentally in animal models and tentatively translated to patients. It should be kept in mind that these processes are evolving at the cellular and subcellular levels before reaching a systemic one.

In his first point, Dr. Palaniyappan correctly notes that oxidative stress is known to be well regulated by negative feedback adaptive responses induced by homeostatic regulation mechanisms of the redox system itself. We agree that in most cases, genetic risk alone is generally well compensated and that additional environmental “hits” during development are required for the emergence of psychiatric symptoms. This is indeed highlighted both in early psychosis patients and animal models [3, 4]. However, depending on the intensity and duration of oxidative stress, the adaptive response and the cellular consequences could be quite different. With a slight and/or short-lived increase of reactive oxygen species (ROS) levels, homeostasis is easily compensated without lasting major cellular consequences. However, in case of a strong and/or long-lasting oxidative stress, compensatory mechanisms can be compromised and redox homeostasis cannot be reestablished, leading to decreased antioxidant defenses and deleterious damage at the levels of membranes, proteins, and DNA [5].

Based on observations in animal models, we propose that additional stress, initiated by subsequent “hits” from baseline genetic risk(s) during sensitive period of development, can overwhelm antioxidant defenses, leading to chronic oxidative stress. This is precisely the situation in which the redox regulatory control, normally maintaining ROS homeostasis, failed to reestablish the physiological set point [5, 6]. Under those conditions, the pathological mechanisms originally involved (neuroinflammation, mitochondria dysfunction or NMDAR hypofunction), would be maintained via complex reciprocal interactions and eventually lead to psychiatric manifestations. As correctly raised by Dr Palaniyappan, there are diverse adaptive and compensatory mechanisms, particularly in early phase of the disease, that may minimize these dysregulations and contribute to heterogeneity across patients. In this respect, we observed that a subgroup of early psychosis patients exposed to childhood trauma but maintaining a relatively preserved cognition, present an upregulation of the TRX/PRX antioxidant system [7].

While the role of a glutathione deficit was at the origin of our hypothesis, it became evident thereafter that other redox regulatory mechanisms could also be involved. A generalized alteration of glutathione levels could be limited to a subgroup of patients or to specific stages of the disease. In other cases, as the oxidative stress seems to be limited to some cell types such as the parvalbumin containing fast-spiking GABA neurons, it is unlikely that they could be detected by MRS, which provides a limited insight into the various cellular and subcellular compartments. Redox homeostasis is well compartmentalized, specifically at subcellular levels (such as membrane, cytosol, mitochondria, nucleus) as well as in spatiotemporal variation of production of redox-signaling molecules: this encompass the NADH/NADPH and the thiol-disulfide redox systems at large (Cys/CySS; GSH/GSSG; Trx/Prx/SRX) [8–10]. This cellular rather than systemic assessment is supported by the recent findings that mitochondria related oxidative stress is present selectively in parvalbumin neurons and that their specific markers are present both in brain and blood of the *gclm-ko* mice and in plasma exosomes of a subgroup from a relatively substantial sample size (*n* = 272) of early psychosis patients [11]. It is thus insufficient and potentially misleading to consider a single parameter (i.e., brain GSH levels as assessed by MRS) and draw conclusion about the state of a complex system such as the redox network. It should also be noted that these MRS studies in patients are often cross-sectional and area based on relatively small underpowered samples.

In his second point, Dr. Palaniyappan questions the feed-forward relationship between NMDA hypofunction and oxidative stress. The data reviewed in Hardingham and Do [12] clearly support the view that the redox response to NMDA receptor activity goes beyond NMDAR-independent ketamine effects. In addition, there are compelling evidence that genetic models of NMDAR hypofunction are prone to oxidative stress. Thus, D-Ser Racemase KO mice mentioned in our review (collaboration with Dr. Joe Coyle) display lasting oxidative stress [4]. Other models, like Grin2A KO mice or mice with GluN1 deletion in forebrain interneurons, are prone to oxidative stress following an environmental challenge, due to a compromised regulation of expression of genes implicated in mitochondrial function and antioxidant defense [13, 14]. Inversely, the evidence that the oxidative stress depresses the NMDAR responsiveness is well documented.

Overall, we propose a cellular circular mechanism that could potentially explain both the generalization of pathological processes such as inflammation, mitochondria, NMDAR and their persistency from early development into adulthood, a pathology initially triggered by a convergence of genetic and environmental risk factors. Such mechanisms may apply to subgroups of patients at various stages of the disease. Mechanism based redox network markers [11, 15] derived from multilevel translational approach are thus much needed for stratification of schizophrenia spectrum heterogeneity.
